# Caveolin-1 Limits the Contribution of BKCa Channel to MCF-7 Breast Cancer Cell Proliferation and Invasion

**DOI:** 10.3390/ijms151120706

**Published:** 2014-11-12

**Authors:** Cheng Du, Li Chen, Haijun Zhang, Zhongchao Wang, Wenchao Liu, Xiaodong Xie, Manjiang Xie

**Affiliations:** 1 Department of Oncology, Xijing Hospital, the Fourth Military Medical University, Xi’an 710032, China; E-Mail: doctorducheng@gmail.com; 2 Department of Oncology, General Hospital of Shenyang Military Area Command, Shenyang 110840, China; 3 Key Laboratory of Aerospace Medicine, Ministry of Education, the Fourth Military Medical University, Xi’an 710032, China; E-Mails: m18591792049@163.com (L.C.); zhjbeijing2008@163.com (H.Z.); 5139005wzc@163.com (Z.W.)

**Keywords:** caveolin-1, large conductance Ca^2+^-activated potassium (BKCa) channel, breast cancer, proliferation, invasion

## Abstract

Increasing evidence suggests that caveolin-1 and large conductance Ca^2+^-activated potassium (BKCa) channels are implicated in the carcinogenesis processes, including cell proliferation and invasion. These two proteins have been proven to interact with each other in vascular endothelial and smooth muscle cells and modulate vascular contractility. In this study, we investigated the probable interaction between caveolin-1 and BKCa in MCF-7 breast cancer cells. We identified that caveolin-1 and BKCa were co-localized and could be reciprocally co-immunoprecipitated in human breast cancer MCF-7 cells. siRNA mediated caveolin-1 knockdown resulted in activation and increased surface expression of BKCa channel, and subsequently promoted the proliferation and invasiveness of breast cancer cells. These effects were attenuated in the presence of BKCa-siRNA. Conversely, up-regulated caveolin-1 suppressed function and surface expression of BKCa channel and exerted negative effects on breast cancer cell proliferation and invasion. Similarly, these opposing effects were abrogated by BKCa up-regulation. Collectively, our findings suggest that BKCa is a critical target for suppression by caveolin-1 in suppressing proliferation and invasion of breast cancer cells. The functional complex of caveolin-1 and BKCa in the membrane microdomain may be served as a potential therapeutic target in breast cancer.

## 1. Introduction

Breast cancer is the second most frequent cancer in the world and is by far the most common cancer in women. A total of 1,665,540 new cancer cases and 585,720 cancer deaths are projected to occur in the United States in 2014 [1]. Despite advances in understanding the causes and treating of primary breast cancer, the 5 year relative survival rate is only about 27% in patients with metastatic disease [2]. Hence, it is of great importance to unravel the molecular mechanisms underlying the progression of this disease.

Caveolin-1, a 21–22 kDa integral membrane protein, is the principal structural component of the non-clathrin, flask-shaped invaginations of the plasma membrane called caveolae [3]. Caveolae are multifunctional membrane microdomains in which caveolin-1 plays direct roles in various cellular events, such as membrane trafficking, cholesterol homeostasis, cell migration and cell cycle [4]. The *caveolin-1* gene (*CAV1*) consists of three exons and localizes to the D7S522 locus in the q31.1 region of human chromosome 7, a known fragile site (FRA7G) that is frequently deleted in human cancer [5]. Available evidence indicates that caveolin-1 is a multifunctional scaffold protein that function, depending on the cellular settings, both as tumor suppressor and promoter [6]. In metastatic prostate cancer cells, caveolin-1 inhibits v-myc avian myelocytomatosis viral oncogene homolog (*c-Myc*)-induced apoptosis and promotes cell survival [7]. In melanoma cell lines, high expression of phospho-caveolin contributes to invasion, migration and increased anchorage independence [8]. On the other hand, oncosuppressive role of caveolin-1 is also reported in recent studies. Specifically, increased expression of caveolin-1 inhibits breast cancer growth and invasiveness in both metastatic MDA-MB-231 and non-metastatic MCF-7 breast cancer cells [9,10,11]. In addition, genetic knockout of caveolin-1 results in mammary gland ductal epithelial cell hyperplasia and accelerates mammary tumorigenesis and lung metastases in mice that is prone to develop breast cancer [10,12,13]. Furthermore, caveolin-1 expression levels are significantly lower in human breast cancer cells than in their normal mammary epithelial counterparts [13]. These studies suggest that caveolin-1 is a tumor suppressor in breast cancer. However, the underlying mechanisms by which caveolin-1 suppresses breast cancer proliferation and metastasis remain understudied.

Large conductance Ca^2+^- and voltage-activated K (BKCa, BK, MaxiK) channels are widely expressed in excitable and non-excitable cells of mammals, and play a variety of functions, including modulation of smooth muscle tone, neuronal firing, synaptic neurotransmitter release, epithelial transport and endocrine cell secretion [14]. The pore-forming α-subunit of BKCa channels is encoded by a single gene named *KCNMA1* (also known as *Slo*). Recent studies demonstrate that *KCNMA1* is amplified in several malignant diseases, including prostate cancer, breast cancer, ovarian and endometrium carcinoma, and contributes to high proliferation rate and malignancy [15,16]. *In vitro* data also indicate that BKCa channels are involved in cell cycle, proliferation, invasion and migration in breast cancer cells [16,17]. BKCa channels interact with various surrounding signaling partners and form cellular environment-dependent functional complexes [18]. For example, BKCa channel is reported to be coupled with Na/K-ATPase in human melanoma IGR39 Cells [19], and with type 3 IP_3_ receptor (IP_3_R3) in breast cancer cell MCF-7 cells [16], regulating cell proliferation. Moreover, BKCa is also demonstrated to be negatively regulated by caveolin-1 in both vascular endothelial cells and smooth muscle cells [20,21]. This macromolecular signaling complex plays an important role modulating vascular contractility. However, whether caveolin-1 interacts with BKCa in breast cancer cells is not known. The functional consequence of this interaction and its impact on breast cancer cell malignancy is also unclear. Therefore, in this study we set out to examine the role of caveolin-1 in modulating the contribution of BKCa channels to breast cancer cell proliferation and invasion.

## 2. Results

### 2.1. BKCa Channels Are Associated with Caveolin-1 in Human Breast Cancer MCF-7 Cells

**Figure 1:**
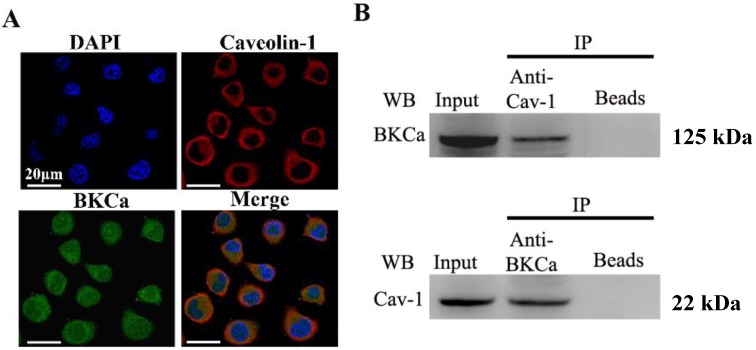
Immunofluorescence and co-immunoprecipitation analysis of BKCa and caveolin-1 in human breast cancer MCF-7 cells. (**A**) MCF-7 cells were immunostained with the anti-BKCa (red) and anti-caveolin (green) antibodies. The nuclei were stained with DAPI (blue). The merged image revealed that BKCa and caveolin-1 are co-localised. Scale bar = 20 μm; and (**B**) Anti-BKCa antibody co-immunoprecipitated caveolin-1 from a total protein lysate prepared from MCF-7 cells. Reciprocally, the anti-caveolin antibody co-immunoprecipitated BKCa. Whole cell lysate was probed for input. Bead lanes contain the protein G conjugated sepharose beads used during the immunoprecipitation without the protein input. All the experiments were repeated three times.

We first examined the possible association of BKCa channels with caveolin-1 in human breast cancer MCF-7 cells. For immunofluorescence analysis, cells were double-labeled with anti-BKCa and anti-caveolin-1 antibodies. Fluorescent images showed there was clear co-localization of BKCa and caveolin-1 in MCF-7 cells (Figure 1A). To determine whether BKCa and caveolin-1 are physically interacted with each other, we performed co-immunoprecipitation using lysis prepared from MCF-7 cells. Typical WB analysis (Figure 1B) showed each protein could be immunoprecipitated by the other one, indicating that both BKCa and caveolin-1 could be part of a common protein complex. By contrast, the negative control, which contained beads used during immunoprecipitation without the protein input, no BKCa or caveolin-1 was precipitated. Taken together, these results indicated that there is close interaction between the BKCa and caveolin-1.

### 2.2. Caveolin-1 Knockdown Results in Activation and Increased Surface Expression of BKCa Channel in MCF7 Cells

**Figure 2:**
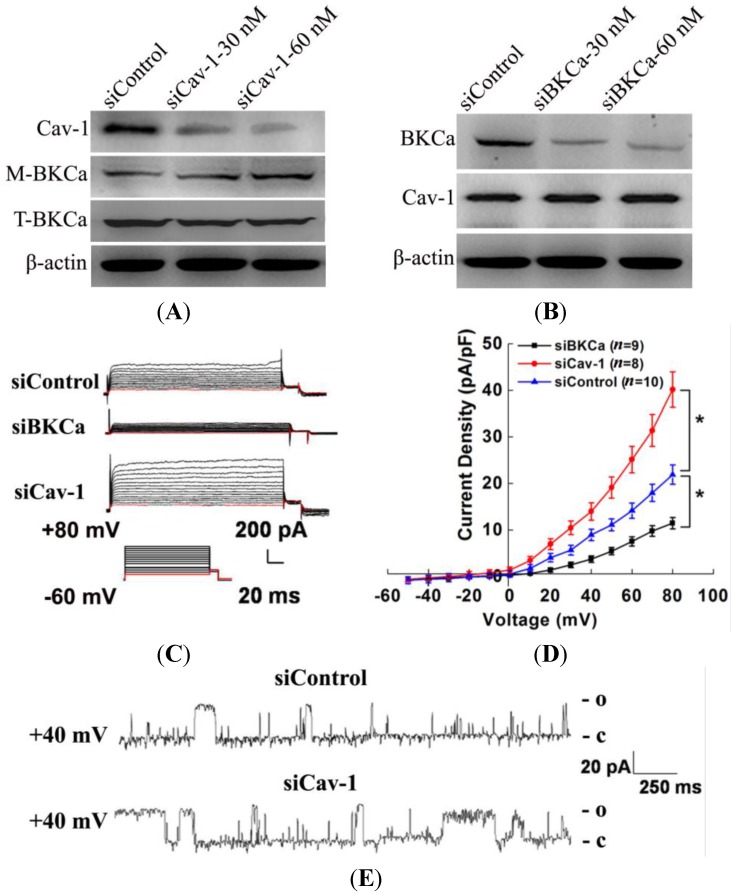

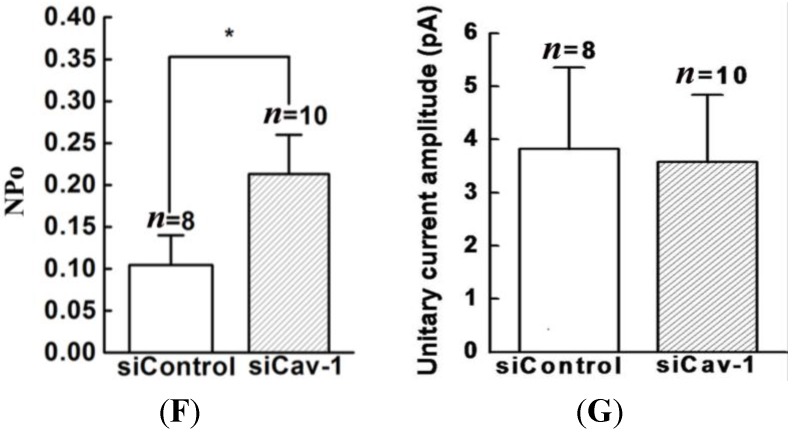
Caveolin-1 knockdown increased the surface expression and activity of BKCa channels in MCF7 Cells. (**A**) MCF-7 cells were transfected with 30 and 60 nM caveolin-1 siRNA (siCav-1) or scrambled siRNA (si Control) for 48 h. Caveolin-1, membrane BKCa (M-BKCa), total BKCa (T-BKCa) and β-actin protein expressions in the cells were analyzed by western blot; (**B**) MCF-7 cells were transfected with 30 and 60 nM BKCa siRNA (siBKCa) or scrambled siRNA (si Control) for 48 h. Caveolin-1, BKCa and β-actin protein expressions in the cells were analyzed by Western blotting; (**C**) Whole-cell K^+^ currents in MCF-7 cells transfected with 30 nM siControl, siBKCa or siCav-1 for 48 h; (**D**) Group data of current-voltage relationships in MCF-7 cells treated with 30 nM siControl (*n* = 10), siBKCa (*n* = 9) or siCav-1 (*n* = 8) for 48 h; (**E**) Representative traces of BKCa single-channel currents in cell-attached patches after the treatment of 30 nM siControl or siCav-1 for 48 h; (**F**) NPo (Po, open probability) was calculated in siControl or siCav-1 transfected MCF-7 cells. siCav-1 increased the NPo significantly; and (**G**) Unitary current amplitude (Am) in BKCa channels were shown against membrane potentials. No significant difference was observed between the two groups. All the experiments were repeated three times. (* *p* < 0.05).

To investigate the interaction between caveolin-1 and BKCa in MCF-7 cells, we knocked down caveolin-1 expression using siRNA and examined its effects on BKCa channel expression and activity. As shown in Figure 2A, the caveolin-1 siRNA (siCav-1) effectively down-regulated the expression of caveolin-1 in a dose-dependent manner. In addition, down-regulated caveolin-1 led to increased membrane expression of BKCa, but the total BKCa expression was not changed. To study whether BKCa can inversely regulate caveolin-1, we knocked down BKCa expression using siRNA. Immunoblotting revealed that BKCa protein level was decreased with the increase of siBKCa, while that of caveolin-1 was not changed (Figure 2B). The quantification of protein levels is shown in the Figure S1. To further confirm the negative regulation of BKCa channels by caveolin-1, we examined the function of BKCa channel in MCF-7 cells under the treatment of 30 nM siCav-1 or siBKCa. Caveolin-1 down-regulation led to a significant increase in whole-cell outward currents (Figure 2C). For example, at the holding potential of −60 mV and the testing potential of +60 mV, the whole cell K^+^ currents were significantly increased by 83% as compared with control. The mean I–V relationships were further expressed in terms of current densities (Figure 2D). Moreover, the inhibitory effects of siBKCa on whole-cell currents further confirmed the effective downregulation of BKCa channel. Single-channel analysis showed that siCav-1 treatment significantly increased the NPo (Po, open probability) of the BKCa channel by 1.1 folds at 40 mV (Figure 2E,F), whereas it did not change unitary current amplitude (Am) (Figure 2G). Collectively, these observations clearly indicated that caveolin-1 down-regulation increased both the expression and activities of BKCa channel.

### 2.3. Caveolin-1 Knockdown Mediated Up-Regulation and Activation of BKCa Channel Promote Breast Cancer Cell Proliferation and Invasion

**Figure 3:**
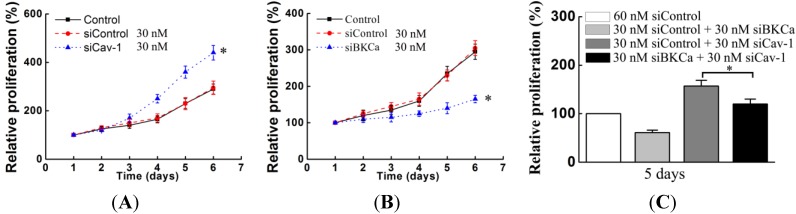

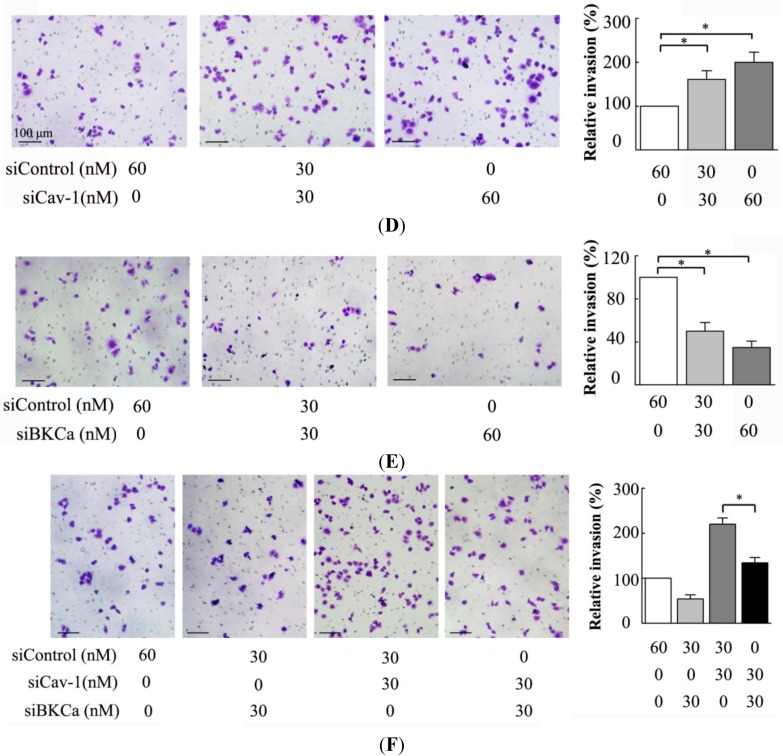
Caveolin-1 knockdown mediated activation of BKCa channel promotes proliferation and invasion in MCF-7 cells. MCF-7 cells were transfected with indicated amounts of siRNAs for 48 h. For MTT assays (**A**–**C**), cells were plated in 96-well plates at an initial density of 4000 cells/well and cultured for the indicated time; For invasion assay (**D**–**F**), cells were plated in 24-well plates at an initial density of 30,000 cells/well and cultured for 24 h. Representative photographs of tumor cells that invaded through a Matrigel-coated filter were taken and shown. The invasive cells were counted in 10 random fields. The experiment was repeated at least three times and results are expressed relative to the number of control. All the experiments were repeated three times. (* *p* < 0.05).

Recent studies suggest that caveolin-1 inhibits breast cancer cell proliferation and invasion [6,10,11]. Furthermore, it is reported that enhanced expression of BKCa contributes to a high proliferation rate and greater invasiveness of breast cancer cells [16,17]. Therefore, we determined the effects of caveolin-1 knockdown mediated up-regulation and activation of BKCa channel on breast cancer proliferation and invasion. First, we knocked down the expression of caveolin-1 and found that the proliferation and invasiveness of MCF-7 cells were significantly increased (Figure 3A,D). Second, we down-regulated the expression of BKCa channel and found that the proliferation and invasion of MCF-7 cells were inhibited (Figure 3B,E). Finally, we co-transfected both siCav-1 and siBKCa into MCF-7 cells and found that siBKCa attenuated the proliferation and invasiveness of caveolin-1-downregulated MCF-7 cells (Figure 3C,F). 

### 2.4. Caveolin-1 Up-Regulation Suppressed Function and Surface Expression of BKCa Channel in MCF7 Cells

**Figure 4:**
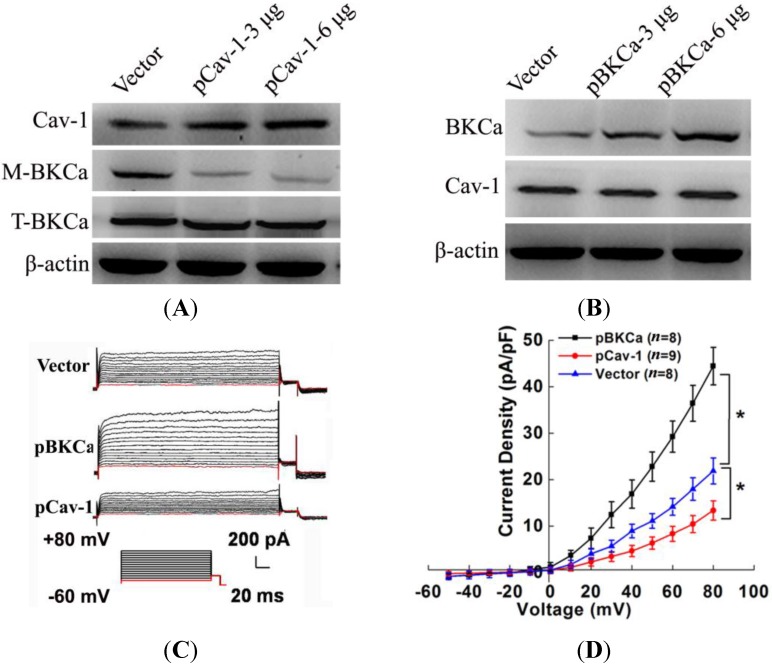

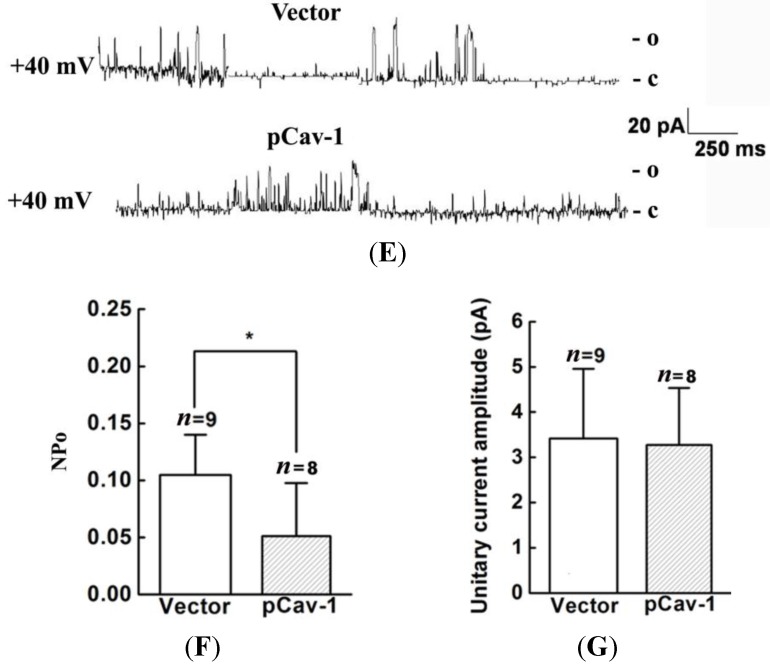
Caveolin-1 overexpression inhibited the expression and activity of BKCa Channels in MCF7 Cells. (**A**) MCF-7 cells were transfected with 3 and 6 μg BKCa plasmid (pBKCa) or control vector for 48 h. Caveolin-1, membrane BKCa (M-BKCa), total BKCa (T-BKCa) and β-actin protein expressions in the cells were analyzed by Western blotting; (**B**) MCF-7 cells were transfected with 3 and 6 μg caveolin-1 plasmid (pCav-1) or control vector for 48 h. Caveolin-1, BKCa and β-actin protein expressions in the cells were analyzed by western blot; (**C**) Whole-cell K^+^ currents in MCF-7 cells transfected with 3 μg pCav-1, pBKCa or vector for 48 h; (**D**) Group data of current-voltage relationships in MCF-7 cells treated with 3 μg pCav-1 (*n* = 9), pBKCa (*n* = 8) or vector (*n* = 8) for 48 h; (**E**) Representative traces of BKCa single-channel currents in cell-attached patches after the treatment of 3 μg pCav-1 or vector for 48 h; (**F**) NPo (Po, open probability) was calculated in pCav-1 or vector transfected MCF-7 cells. pCav-1 decreased the NPo significantly ; and (**G**) Unitary current amplitude (Am) in BKCa channels were shown against membrane potentials. No significant difference could be observed between the two groups. All the experiments were repeated three times. (* *p* < 0.05).

Because our data have shown that caveolin-1 inhibition in MCF-7 breast cancer lines promote cell proliferation and invasion via activation and up-regulation surface expression of BKCa channels, we next investigated whether enforced caveolin-1 expression may lead to opposite effects. We transfected the plasmids of caveolin-1 (pCav-1) or BKCa (pBKCa) into MCF-7 cells and found that caveolin-1 overexpression significantly decreased the membrane expression of BKCa channels in a dose-dependent manner (Figure 4A), whereas the total BKCa expression was not changed. Moreover, up-regulated BKCa showed no effects on the expression of caveolin-1 (Figure 4B). The quantification of protein levels is shown in the Figure S2. Our electrophysiological analysis revealed that treatment of 3 μg pCav-1 resulted in a significant decrease in whole-cell outward currents (Figure 4C). For example, with a holding potential of −60 mV and a testing potential of +60 mV, the whole cell K^+^ currents were significantly decreased by 41% as compared with control. The mean I–V relationships were further expressed in terms of current densities (Figure 4D). Furthermore, the enhanced whole-cell currents by pBKCa further confirmed the effective upregulation of BKCa channel. Single-channel analysis showed that pCav-1 treatment significantly decreased the NPo (Po, open probability) of the BKCa channel by 50% at 40 mV (Figure 4E,F), whereas it did not change unitary current amplitude (Am) (Figure 4G). These results confirmed that caveolin-1 exerts a negative regulatory effect on BKCa channel function.

**Figure 5:**
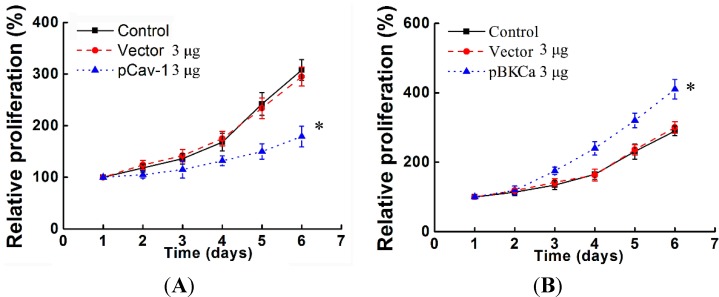

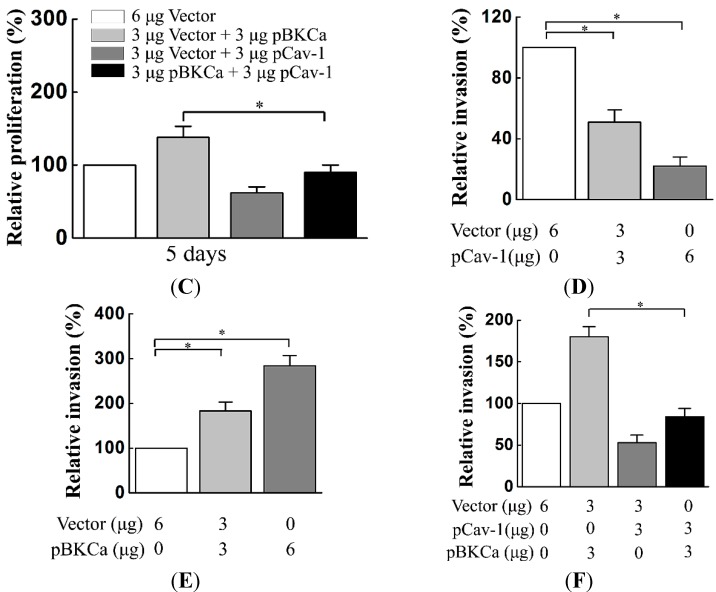
Caveolin-1 upregulation suppressed proliferation and invasion via decreasing expression and function of BKCa channel in MCF7 cells. MCF-7 cells were transfected with indicated amounts of plasmids for 48 h. For MTT assays (**A**–**C**), cells were plated in 96-well plates at an initial density of 4000 cells/well and cultured for the indicated time. For invasion assay (**D**–**F**), cells were plated in 24-well plates at an initial density of 30,000 cells/well and cultured for 24 h. The invasive cells were counted in 10 random fields. The experiment was repeated at least three times and results are expressed relative to the number of control (100%). All the experiments were repeated three times. (* *p* < 0.05).

### 2.5. Inhibition of BKCa Channel by Caveolin-1 Up-Regulation Suppressed Breast Cancer Cell Proliferation and Invasion

To determine whether caveolin-1 over-expression would affect cell proliferation and invasion via BKCa channels, we also transfected caveolin-1, BKCa or a combination of both into MCF-7 cells. The results indicated that overexpression of caveolin-1 alone inhibited the proliferation and invasion of MCF-7 cells (Figure 5A,D). Conversely, overexpression of BKCa promoted the proliferation and invasiveness of MCF-7 cells (Figure 5B,E). Interestingly, co-transfection of caveolin-1 abrogated the proliferation and invasiveness of BKCa overexpressing MCF-7 cells (Figure 5C,F). These data indicated that caveolin-1 inhibition of cell proliferation and invasion is at least partially dependent on suppression of BKCa.

## 3. Discussion

The present study indicated that caveolin-1 and BKCa channel were co-localized and could be reciprocally co-immunoprecipitated in human breast cancer MCF-7 cells. We also demonstrated that caveolin-1 knockdown resulted in activation and increased surface expression of BKCa channels, and subsequently promoted the proliferation and invasiveness of breast cancer cell. These effects were attenuated in the presence of siBKCa. Conversely, up-regulated caveolin-1 suppressed function and surface expression of BKCa channels and exerted negative effects on breast cancer cell proliferation and invasion. Similarly, these opposing effects were abrogated by BKCa up-regulation.

Caveolae/lipid rafts function as molecular hubs and platforms, integrating the activity of a multitude of signaling molecules, such as tyrosine kinase receptors, phosphatases and endothelial nitric oxide synthase (eNOS) [3,22,23]. As the essential constituent protein of caveolae, caveolin-1 is implicated in the integration of numerous signaling pathways that are temporally and spatially controlled [23,24]. A substantial number of detailed analyses suggest that caveolin-1 may enhance cell survival and migration, notably in prostate cancer cells [7,25,26]. On the other hand, increasing evidence demonstrates that caveolin-1 suppresses the transformed phenotype of cancer cell. For example, caveolin-1 was reported to inhibit proliferation, anchorage-independent colony formation and matrix invasion in breast cancer [6,9,11]. Consistent with previous studies, our findings suggested that caveolin-1 negatively regulate proliferation and invasion in human breast cancer MCF-7 cells. Caveolin-1 seems to demonstrate both tumor suppressive and oncogenic activity depending on the cellular settings. However, the oncogenic and tumor suppressor function of caveolin-1 have been reported to occur within a tumor type. This contradictory action of caveolin-1 underscores the complexity and importance of unraveling the mechanism by which it acts as a breast cancer modulator.

BKCa channels are expressed in both normal and cancerous cells. The BKCa channel encoding gene KCNMA1 is amplified in about 16% of late-stage prostate cancers and in about 1.9% of breast cancers [15,16]. This amplification is significantly associated with high tumor grade and poor tumor-specific survival. *In vitro* studies proved that knockdown of KCNMA1 inhibited cell proliferation or invasiveness in different types of cancer, such as glioblastoma, breast and prostate cancer, although this not seemed to apply to all [16,27,28]. How the BKCa channel promotes proliferation or invasion of cancer cells is not completely illuminated. It is proposed that BKCa channels are involved in cancer cell migration presumably by contributing to the plasma membrane ionic fluxes that underscore cell volume regulation, particularly as these cells alter their volume through the restricted intercellular spaces available to tumor cells [29]. In a recent study by Abdallah Mound *et al.*, siBKCa decreased both cyclin-D1 and cyclin-dependent kinase 4 expression in MCF-7 cells, which led to inhibited proliferation [30]. In line with these findings, the present study also demonstrated that down- or up-regulation of BKCa resulted in inhibition or promotion of breast cancer proliferation and invasion.

As is known that BKCa channels are part of macromolecular signaling complexes that include receptors, kinases, ion channels and other catalytically active molecules to mediate local and spatially directed signaling [14,31,32]. Regarding this context, we have been intrigued by recent reports showing that BKCa channels are targeted to caveolae microdomains in endothelial and smooth muscle cells [20,21]. Whether a functional interaction between caveolin-1 and BKCa exists in breast cancer cells and its impacts on cell malignancy are not known. To this end, we chose the human breast cancer MCF-7 cell line that was frequently used in studies about caveolin-1 and BKCa. We found that caveolin-1 and BKCa are co-localized in MCF-7 cells. Moreover, caveolin-1 and BKCa can be immunoprecipitated reciprocally, implying a potential interaction between these two proteins. It has been proven that caveolin-1 exerts a negative regulatory effect on BKCa channel functions in bovine aortic endothelial cells and freshly dissociated myocytes of rat aorta [20]. Thus, we genetically modified the expression of caveolin-1 and found that the whole-cell current of BKCa was negatively modulated. In addition, single-channel analysis showed that the open probability of the BKCa channel was also inversely regulated by modified caveolin-1 expression, whereas the unitary current amplitude was not changed. These results were consistent with previous reports that caveolin-1 co-expression downsized the macroscopic currents generated by expression of BKCa alone in HEK293T cells, and changed the mean I V curves of current density for BKCa [20]. To dissect the mechanism of this effect, we detect the expression of BKCa. Immunoblotting showed that the total expression of BKCa was unaffected; interestingly, the cellular membrane expression was inversely regulated by down- or up-regulation of caveolin-1. Our findings are consistent with previous reports showing that knocking down caveolin-1 protein by siRNA resulted in increased functional BKCa current/channel density at the surface membrane [32,33]. However, the mechanism by which caveolin-1 exerts negative effects on BKCa function and expression remain unclear. Someone proposed that caveolin-1 constitutively down-regulate BKCa surface expression [19,32]. Others argued this effect was possibly due to that caveolin-1 retained BKCa in intracellular compartments [20,21]. To uncover the exact mechanism, further studies are needed.

We next examined the functional impact of the interaction between caveolin-1 and BKCa channels on breast cancer cells. The results indicated that caveolin-1 knockdown mediated up-regulation and activation of BKCa channel promote cell proliferation and invasion. These effects were attenuated in the presence of siBKCa-. Conversely, inhibition of BKCa channel by caveolin-1 up-regulation exerted negative effects on breast cancer cell proliferation and invasion. Similarly, this opposing effect was abrogated by BKCa up-regulation. Together, these results suggested that the caveolin-1 limited the contribution of BKCa channel on breast cancer proliferation and invasion.

There are some limitations in our study. Although we proved the interaction between BKCa and caveolin-1 in MCF-7 cells, whether our findings can be extended to other cancer cell contexts is yet to be verified. Additionally, these findings further require validation in biological tumor models using cell lines stably-transfected with BKCa/caveolin-1. Thirdly, given that other molecules (e.g., IP3R, Ca^2+^ and Na/K-ATPase) can interact with BKCa [19,30], we cannot exclude the possibility that these potential molecules may affect the interaction between BKCa and caveolin-1 without further study. Finally, BKCa channel activity is reported to be affected by cholesterol [28], whether caveolin-1 negatively regulates membrane expression and function of BKCa via modulating membrane cholesterol should be addressed in future study.

In conclusion, our results suggest that caveolin-1 plays an important role in breast cancer proliferation and invasion by regulating the surface expression and activation of BKCa. These novel findings raise the intriguing possibility that the functional complex formed by caveolin-1 and BKCa in the membrane microdomain may represent a new mechanism of various phenotypic changes that are associated with breast cancer malignancy and thus may be served as a potential therapeutic target.

## 4. Materials and Methods

### 4.1. Cell Culture

Human breast cancer MCF-7 cells were obtained from the American Type Culture Collection (ATCC, Manassas, VA, USA) and were cultured in Dulbecco’s modified Eagle’s medium (HyClone, Logan, UT, USA) supplemented with 10% heat-inactivated fetal bovine serum (HyClone, Logan, UT, USA) at 37 °C in a humidified incubator with 95% air and 5% CO_2_.

### 4.2. Immunofluorescence Microscopy

MCF-7 cells were grown on glass cover slips, fixed with ice-cold methanol, then permeabilized with 0.1% Triton X-100 in phosphate buffered saline (PBS). After incubation with 1% bovine serum albumin (BSA) in PBS, cells were incubated overnight at 4 °C with a mouse anti-caveolin-1 antibody (1:100, Abcam, Cambridge, MA, USA) plus a rabbit anti-BK-α antibody (1:100, Alomone, Jerusalem, Israel). Subsequently, cells were washed in PBS and incubated with fluorescein isothiocyanate-conjugated goat-anti-mouse secondary antibody or Texas Red-conjugated goat-anti-rabbit secondary antibody (1:400, Abcam) for 1 h at 37 °C. Cell nuclei were counter stained with 4',6-diamidino-2-phenylindole dihydrochloride (DAPI) solution (Sigma, St. Louis, MO, USA).

### 4.3. Co-Immunoprecipitation

Immunoprecipitation was performed as previously described with modifications [20]. Briefly, cells were lysed in lysis buffer (150 mM NaCl, 50 mM Tris-HCl,100 mM NaF, 5 mM EDTA, 1 mM Na_3_VO_4_, 0.5 mM iodoacetamide, 10 mM 4-(2-hydroxyethyl)-1-piperazineethanesulfonic acid (HEPES), pH7.4, 0.1% NP-40, 0.25% sodium deoxycholate, plus protease inhibitors). The homogenates were centrifugated at 4000× *g* for 10 min at 4 °C. The supernatant was precleared by incubation for 2 h with 50 μL protein-*G*-agarose beads and centrifuged (15,000× *g*, 30 min, 4 °C). Precleared supernatants were incubated with 2 μg of polyclonal anti-caveolin-1 or anti-BK-α antibodies at (4 h, 4 °C), followed by the addition 25 μL of protein-*G*-Sepharose beads (overnight). The immune complexes were centrifuged (2000× *g*, 5 min). The beads were washed (10% glycerol/lysis buffer), eluted in sample buffer, boiled, and subjected to Western blot.

### 4.4. Plasmids and siRNAs Transfection

The plasmids pIRES-BKCa was a gift from J. D. Lippiat (University Laboratory of Physiology, Oxford, UK) [34]. The open reading frame of human caveolin-1 was cloned into the mammalian plasmid pCMV-Neo (GeneCopoeia, Rockville, MD, USA). siRNA sequences targeting BKCa channel and caveolin-1 were as follows: BKCa, 5'-gtgggtctgtccttccctact-3'; Caveolin-1, 5'-agacgagcugagcgagaagcatt-3' (Genechem, Shanghai, China). The scrambled siRNA was used in the control group. The corresponding sequences were as follows: BKCa, 5'-ggcccttcgtatgcgctcttt-3'; caveolin-1, 5'-gagaggcatcgacctgtaagaga-3' (Genechem, Shanghai, China). Transfection was performed by Lipofectamine 2000 (Invitrogen, Carlsbad, CA, USA) as described previously [11]. Cells were transfected with plasmid or siRNA at different doses as indicated for 48 h before functional assays were carried out.

### 4.5. Western Blot

Proteins were extracted with the M-PER mammalian protein extraction reagent added with protease inhibitors (Thermo, Rockford, IL, USA). Supernatants were collected and the protein was quantified using the bicinchoninic acid (BCA) protein assay kit (Thermo, Rockford, IL, USA). Membrane protein was isolated using the Mem-PER Membrane Protein Extraction kit (Thermo) according to the protocols. Proteins were separated using NuPAGE 4%–12% Bis-Tris gel (Invitrogen, Carlsbad, CA, USA), and transferred to polyvinylidene fluoride (PVDF) membranes (Millipore, Billerica, MA, USA). The proteins were detected with the following antibodies: Rabbit anti-BKCa polyclone antibody (1:600, Alomone, Jerusalem, Israel), rabbit anti-caveolin-1 polyclone antibody (1:1000, Abcam).The primary antibodies were detected with horseradish peroxidase-conjugated secondary antibodies (1:15000, Abcam), and the signals were developed using an enhanced chemiluminescence detection kit (Thermo).

### 4.6. Patch Clamp

Patch clamp was performed as previously described [35]. Whole-cell and single-channel of BKCa currents were recorded with an amplifier (CEZ-2300, Nihon Kohden Co., Tokyo, Japan) and a version interface (Axon Instruments, Foster City, CA, USA) as reported previously. Whole-cell BKCa currents were recorded with the conventional voltage clamp configuration. Current densities were obtained by normalizing currents to the cell membrane capacitance (Cm). The extracellular (bath) solution contained 135 mM NaCl, 5.0 mM KCl, 1.8 mM CaCl_2_, 1.0 mM MgCl_2_, 10 mM HEPES, 10 mM glucose, and 5.0 mM 4-aminopyridine (4-AP), equilibrated with 95% O_2_ and 5% CO_2_ at pH 7.4 adjusted by NaOH. 4-AP in the bath solution was used to exclude the interference from voltage-dependent K^+^ (KV) channel currents. The internal (pipette) solution contained 50 mM KCl, 70 mM K-Asp, 8.0 mM NaCl, 2.0 mM MgCl_2_, 1.0 mM Na_2_ATP, 0.5 mM GTP, 10 mM HEPES, 1.0 mM CaCl_2_, 2.0 mM EGTA equilibrated with 95% O_2_ and 5% CO_2_ at pH 7.2 titrated with KOH. Single-channel currents of BKCa were recorded in cell-attached membrane patches. The pipette (external) solution contained 40 mM K-Asp, 100 mM KCl, 1.0 mM CaCl_2_, 10 mM HEPES equilibrated with 95% O_2_ and 5% CO_2_ at pH 7.4 titrated with KOH. The bath solution contained 100 mM K-Asp, 40 mM KCl, 10 mM HEPES, 2.0 mM EGTA, 1.98 mM CaCl_2_ equilibrated with 95% O_2_and 5% CO_2_ at pH 7.4 titrated with KOH.

### 4.7. MTT Assays

Breast cancer cells were plated in the 96 well plates at a density of 3000 cells per well and cultured for 24 h. At the end of culture for each indicated time points, cells were incubated with MTT (5 mg/mL) for 4 h at 37 °C and treated with dimethyl sulfoxide (DMSO) (150 μL/well) to dissolve the purple formazan crystals formed. The absorbance was read at 570 nm using a micro-plate reader (Bio-Tek Instruments, Inc., Winooski, VT, USA).

### 4.8. Invasion Assays

Invasion assays were carried out as described previously by using growth factor reduced insert plates coated with Matrigel (BD Biosciences, Bedford, MA, USA) [11]. The bottom chamber was filled with 600 μL DMEM containing chemoattractant (10% FBS). Three × 10^4^ cells suspended in 200 μL serum free medium were plated in the upper chamber and incubated at 37 °C for 24 h. Non-invading cells were scrubbed with a cotton-tip swab. The cells that penetrated through the filter were stained with crystal violet and counted using a phase-contrast microscope at a magnification of ×200 in 10 randomly selected fields, and the mean number of cells per field was recorded.

### 4.9. Statistical Analysis

Data were presented as mean ± standard deviation. Differences between the means were determined by two-tailed Student’s *t*-test. Two-sided *p* values less than 0.05 were considered statistically significant.

## 5. Conclusions

BKCa channels are associated with caveolin-1 in human breast cancer MCF-7 Cells. Caveolin-1 limits the contribution of BKCa on breast cancer cell proliferation and invasion by negatively regulating its function and surface expression. The functional complex of caveolin-1 and BKCa may serve as a new therapeutic target in breast cancer.

## Supplementary Materials

Supplementary figures can be found at http://www.mdpi.com/1422-0067/15/11/20706/s1.
